# 

**Published:** 1986-12

**Authors:** 


**Editor**
Mr M. G. Wilson
*40 Coombe Lane, Bristol BS9 2BJ*
*Tel: 0272-682320*

**Assistant Editor**
Dr P. R. Goddard

**Editorial Committee**
Mr B. P. Jones (Secretary),
*Medical Librarian, Bristol University*
Dr J. L. Burton

**Correspondents**
Dr A. E. Adam
Dr J. D. Davies
Dr J. Jancar
Dr H. G. Mather
Professor G. M. Stirratt
Dr J. L. G. Thomson
Dr M. J. Whitfield
Professor G. K. Wilcock
Professor R. C. N. Williamson
Dr D. W. Wright

**BRISTOL MEDICO-CHIRURGICAL SOCIETY**

**President**
Mr H. B. Griffith

**Secretary**
Mr R. N. Baird,
*23 Old Sneed Park, Bristol BS9 1RG*

**Treasurer**
Dr N. C. Tricks,
*The Mill, Wrington, Bristol*

**Published by**
**Hermiston Publications,**
*BMA Scottish House,*
*7 Drumsheugh Gardens,*
*Edinburgh EH3 7QP*

**Production Manager**
June Macnab

**Typesetting by**
*Avon Communications Ltd.,*
*Avon House, Nailsea, Bristol BS19 2DJ*

**Printed by**
*Scottish County Press, Sherwood Industrial Estate,*
*Bonnyrigg, Midlothian*

Established 1883

### BRISTOL MEDICO-CHIRURGICAL JOURNAL

**DECEMBER 1986**
**Volume 101 (vi)**

Published bi-monthly and distributed free of charge to all doctors in the South West of England. Overseas subscription £12 per annum.

Scire est nescire, nisi id me alius sciret.
*Lucilius (180–102 BC)*

### BRISTOL MEDICINE

**Bristol Medicine acknowledges with gratitude the support and encouragement it has received from Bristol-Myers Pharmaceuticals**

### Index for 1986

Abrams P. H. 71.
Acute-phase response. Spence C. E. 42.
Algodystrophy. Churcher M. 19.
Aldridge S. 109.
Al Zam S. Z. 30, 41.
Ammonites revisited. Williamson R. C. N. 16.
Angioendotheliosis, neoplastic. Rutherford G. S., Brownell D. E. 41.
Ano-rectal incontinence, postnatal repair for. Mortensen N. J. C. 22.
Aorto-enteric fistulae, management of. Thomas W. E. G., Baird R. N. 68.
Appendicitis in Bristol – 100 years ago. Cooper M. J. 126.
Ascending Aorto-bifemoral grafts. Wilmshurst C. C. et al., Gardner A. M. N., Pagliero K. M. 19.
Aspirin dose problem is now solved. Eastham R. D. 42.
Baird R. N. 68, 70.
Baker A. 94.
Barritt D. W. 101.
Bile duct stones, endoscopic papillotomy in the management of. Thomson M. H. 70.
Bladder, management during and after abdominal surgery. Bristol J. B. 22.
Bone scanning in painful conditions of the feet. Maurice H. 94.
Book Reviews –
Brain CT. An introduction. J. R. Bradshaw 92.
Citadel of the Senses. MacDonald Critchley 116.
Cuisine of Hungary. Lang G. 44.
Dictionary of computing. 99.
Essentials of Dermatology, Burton J. L. 44.
Pathology in Surgical Practice. Hadfield, Hobsley and Morson. 118.
Seamarks, their history and development. John Naish. 118.
Urinary tract and the catheter. N. Slade, Gillespie W. A. 116.
Brachial Plexus lesions. Parry C. B. W. 94.
Breast, benign disease of in Torbay. Hughes R. G. 20.
Breast, postoperative care of. Griffiths D. A. 20.
Briggs J. C. 30.
Bristol Eye Hospital – an historical perspective. Marmion V. J. 134.
Bristol J. B. 22.
Bristol Medico-Historical Society – Proposal to form a new Society. Wilson M. G., Sluglett J. 23.
Brown D. F. J. 42.
Brownell D. E. 41.
Buirski G. 33.
Bunker T. D. 94.
Burns-Cox C. J. 39, 105.
Burton J. L. 15, 59, 135.
Calder S. J. 6.
Channer J. L. 41, 42.
China, health care in today. Williams A., Jones E. 114.
Chronic venous leg ulcers and the role of dressings in their treatment. Calder S. J., Leaper D. J. 6.
Churcher M. 19.
Clyne C. A. 19.
Colon, undifferentated carcinomas of. Al Zam S. J., Davies J. D. 41.
Colonic Lavage, antegrade, in acute colonic obstruction. Foster M. E., Johnson C. D. 56.
Cooper M. J. 70, 126.
Coronary angioplasty, percutaneous transluminal, the Bristol experience of the first 32 cases. Aldridge S., Papouchadou M., Walker P., Vann-Jones J., Wilde P. 109.
Coronary patients, do we tell them enough? Trelawney Ross C. P., Jordan S. C. 106.
Davies J. D. 30, 41, 41, 42.
Davis P. 105.
Dankwa E. K. 41.
Deaths in Anguilla, Burns. Cox C. J. 39.
Diabetes mellitus and mental handicap. Jancar J., Lansdall Welfare R. W. 82.
Dimethyl Sulphoxide in the treatment of Interstitial Cystitis. Parivar F. 20.
Dipyramidole and Aspirin Therapy in the prevention of early failure in Femoro-distal bypass grafts. Clyne C. A. 19.
Distraction, displacement and alarm. Radford P. 145.
Dowling A. 88.
Dual chamber pacemakers. Sanderson J. E. 40.
Duodenal ulcer, rational prescribing for. Williams J. G. 39.
Eastham R. D. 42.
Endoscopy, upper gastro-intestinal in a general surgical unit. Johnson C. D. 83.
Eruptive Vellus Hair Cysts. Grattan C. E. H., Harman R. R. M. 10.
Feneley R. C. L. 129.
Foot Pump. Gardner A. M. N. 20.
Foster M. E. 56.
Gallstones, are they preventable? Heaton K. W. 70.
Gardening – the great test of health. Steen T. R. 66.
Gardner A. M. N. 20, 19.
Gillatt D. A. 71.
Gingell J. C. 71.
Goddard P. 33, 38.
Gorman W. P. 58.
Grattan C. E. H. 10.
Green Paper, an hypothesis to explain the delay in its appearance. Dowling A. 88.
Griffith H. 29, 148.
Griffiths D. A. 20.
Griffiths J. 36.
Gun Money. Goddard P. 38.
Haemophilia, learning about. Kendrick C. 132.
Hall G. H. 40.
Halpin D. S. 20.
Harman R. R. M. 10.
Health Care in China today. Williams A., Jones E. 115.
Heaton K. W. 70.
Hip, hemiarthroplasty of. Baker A. 94.
Histopathological Audit, an approach to. Smith J. H. F., Al Zam S. Z., Briggs J. C., Davies, J. D. 30
Hughes R. G. 20.
Hughes-Games J. 147.
Hypertension, appreciating, quicksands and firm ground. Barritt D. W. 101.
Imtran Revolution. Griffith H. 29.
Intestinal blood flow and absorption. Mountford R. H. 40.
Jancar J. 53, 79, 82.
Jeyasingham K. 22, 68.
Johnson C. D. 56, 83.
Jones E. 115.
Jordan S. C. 106.
Keen G. 68.
Kendrick C. 132.
Kidney stone surgery, a second class service? Gingell J. C. 71.
Leaper D. J. 6.
Lennard M. B. 90, 147.
Letters to the Editor –
Griffith H. 146 (?).
Harman R. R. M. 46.
Pardoe R. B. 46.
Kennedy C. 46.
Bracey D. J. 87.
Levet, Dr Robert: an obscure practiser in physic. Griffiths J. 36.
Library services – a computer terminal. Rickett J. W. S. 22.
Lymph nodes, necrotic and their connective tissue framework. Channer J. L., Davies J. D. 42.
McMaster P. 86.
Marmion V. J. 134.
Maurice H. 94.
May R. E. 71.
Measurement of Glycosylated Haemoglobin, factors affecting. Hartog M. 39.
Memorable Membership Moment, A. Burton J. L. 59.
Mental Handicap in Bristol and Bath, the history of. Jancar J. 53, 79.
Microvascular surgery with a review of a personal series of 75 reconstructive cases. Townsend P. L. G. 71.

Moore R. E. 42.
Mountford R. H. 40.
Murdoch, Iris, an evening with. Wilson M. G. 91.
Muscat I. 41.
Myelitis, Transverse, and myelography. Rowe P. A., Gorman W. P. 58.
Myocardial infarction, Assessment of opinions on personalised advice sheet. Burns-Cox C., Davies P., Teague S. 105.
Neurogenic Claudication. Halpin D. S. 20.
No fault compensation for accidental injury. Stirrat G. M. 77.
Obituary –
Michael Lennard 90.
Oesophageal function studies before and after surgery. Jeyasingham K. 68.
Oesophageal Lengthening V–Y Gastroplasty with partial or total fundoplication for acquired short oesophagus. Jeyasingham K. 22.
On a bicycle made for two. Troughton A., Taylor R. 95.
Opening of the Monica Britton Exhibition Hall of Medical History. Wilson M. G. 17.
Pagliero K. M. 19.
Pancreas transplantation. McMaster P. 68.
Paper Weights, glass. Ross F. G. M. 141.
Papouchado M. 109.
Parivar F. 20.
Parry C. B. W. 94.
Pleural Lipoma, CT diagnosis of. Buirski G., Goddard P. 33.
Poet's Corner 147.
Pokhara, Bus 239 to. Burton J. L. 135.
Pyomyositis, A case of. Muscat I. 41.
Radford, P. 145.
Rectal biopsies, spurious smooth muscle tumours in. Dankwa E. K., Channer J. L., Davies J. D. 41.
Rickett J. W. S. 22.
ROC Curves: aids to diagnosis. Hall G. H. 40.
Roskovec A. 18.
Ross F. G. M. 141.
Rowe P. A. 58.
Rutherford G. S. 41.
Shoulder arthroscopy, results in. Bunker, T. D. 94.
Silastic particles and malignancy. Winson M. I. 94.
Sinus node disease, ischaemia and. Shaw D. B. 39.
Skins are simpler than you think. Burton J. L. 15.
Sluglett J. 23.
Smith J. H. F. 30.
South West Orthopaedic Club 94.
Spence C. E. 42.
Spinal cord damage during surgery of the thoracic aorta, prevention of. Keen G. 68.
Spleen, the injured, is there a place for conservation? Cooper M. J., Williamson R. C. N. 70.
Splenectomy for the physician. White H. J. O. 70.
Staphylococcus, testing of coagulase negative. Moore E. P., Brown D. F. J. 42.
Starting to sail. Rozkovec A. 18.
Steen E. 64.
Steen T. R. 66.
Stirrat G. M. 77.
Surgical Club of S.W. England 19, 68.
Taylor R. 95.
Teague S. 105.
Thomas W. E. G. 68.
Thomas M. H. 70.
Thyroiditis, lymphoid, the clinical spectrum of. Webb A. J. 71.
Townsend P. L. G. 71.
Trelawney-Ross C. P. 106.
Troughton A. 95.
Urinary peak flow measurement in prediction of acute retention following abdominal surgery. Gillatt D. A., May R. E., Abrams P. H. 71.
Urological Opprobria, the assessment of. Feneley R. C. L. 129.
Vann-Jones J. 109.
Walker P. 109.
Webb A. J. 71.
Welfare R. W. L. 82.
West country physicians Club 39.
Wessex Branch of the Association of Clinical Pathologists. 41.
Wilde P. 109.
Williams A. 115.
Williams J. G. 39.
Williamson R. C. N. 16, 70.
Wilmshurst C. C. 19.
Wilson M. G. 17, 23, 91, 147.
Witch Doctor, the resurgence of in post colonial Africa. Steen E. 64.

